# Molecular aspects of the teratogenesis of rubella virus

**DOI:** 10.1186/s40659-019-0254-3

**Published:** 2019-08-28

**Authors:** Suji George, Rajlakshmi Viswanathan, Gajanan N. Sapkal

**Affiliations:** Diagnostic Virology Group, ICMR-National Institute of Virology, 20-A, Dr. Ambedkar Road, Pune, Maharashtra 411001 India

**Keywords:** Rubella, Teratogenesis, Apoptosis, Mitochondria

## Abstract

Rubella or German measles is an infection caused by rubella virus (RV). Infection of children and adults is usually characterized by a mild exanthematous febrile illness. However, RV is a major cause of birth defects and fetal death following infection in pregnant women. RV is a teratogen and is a major cause of public health concern as there are more than 100,000 cases of congenital rubella syndrome (CRS) estimated to occur every year. Several lines of evidence in the field of molecular biology of RV have provided deeper insights into the teratogenesis process. The damage to the growing fetus in infected mothers is multifactorial, arising from a combination of cellular damage, as well as its effect on the dividing cells. This review focuses on the findings in the molecular biology of RV, with special emphasis on the mitochondrial, cytoskeleton and the gene expression changes. Further, the review addresses in detail, the role of apoptosis in the teratogenesis process.

## Background

Rubella or German measles, caused by rubella virus (RV), was described for the first time by two German physicians in the mid-eighteenth Century [1]. Although initially it was thought to be a mild disease infecting children; in 1941, an Australian ophthalmologist, Norman McAlister Gregg noticed, that infants born with congenital cataracts also had congenital heart disease [2]. Most of the mothers of these infants had rubella infection during the first trimester of pregnancy. He correlated the occurrence of rubella with genetic defects in infants for the first time. Later, similar observations were also made by others [3–5]. Thus, the most serious effect of RV infection is its teratogenicity [1, 6, 7]. The birth defects seen in infants include blindness, deafness, congenital heart disease, mental retardation and neurological complications, all of them collectively referred to as congenital rubella syndrome (CRS) [8].

**Fig. 1:**
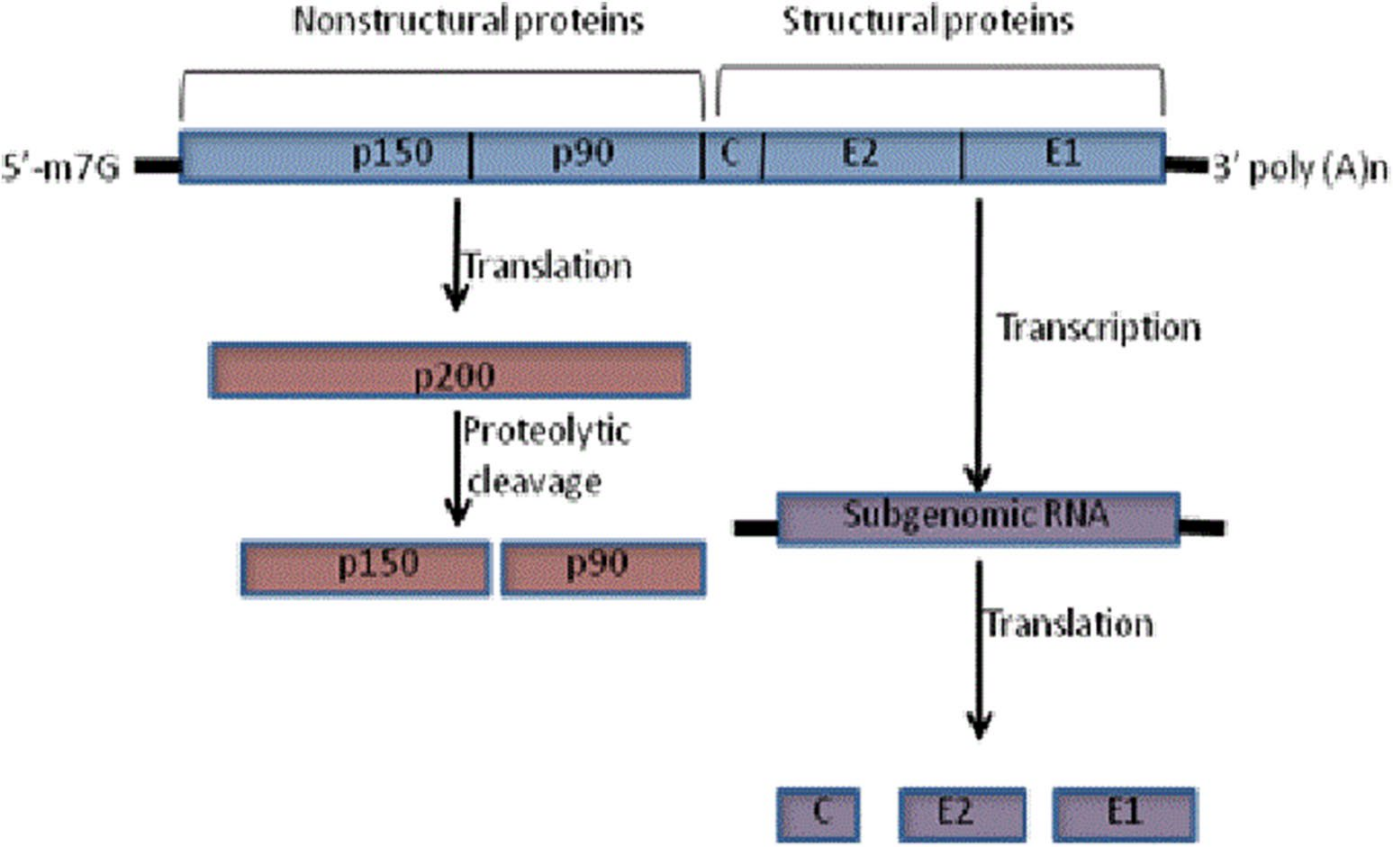
Schematic diagram showing translational processing of non structural and structural proteins of rubella. The rubella genome consists of two non overlapping ORF, the 5′ ORF codes for the non-structural proteins and 3′ ORF codes for the structural proteins. The 5′ ORF is translated to poly protein precursor p200 which is then cleaved to produce the non-structural proteins p150 and p90. The 3′ ORF is translated to poly protein precursor p100, which subsequently undergoes post translational modification to form the final mature capsid (C) and envelop protein (E1 and E2)

RV is a member of the rubivirus genus of the *togaviridae* family [9]. The genome of the virus is approximately a 10 Kb long, positive sense single stranded RNA. The virion consists of a spherical core, composed of a capsid protein and a single copy of the RNA genome. The core is covered by host derived lipid bilayer containing 5 to 6 nm spikes which protrude from the virion surface. The viral genome codes for the two non structural (p90, p150) and three structural proteins (C, E1, and E2). The genomic RNA serves as a template for the translation of the non-structural proteins, which are synthesized in the form of a precursor (p200) that is further cleaved by the protease activity of p150 protein to form p150 and p90. The two non-structural proteins (p150 and p90), then synthesize subgenomic mRNA which is subsequently required for the synthesis of the viral capsid protein (C) and surface glycoproteins (E1 and E2) (Fig. 1).

## Attachment and entry of the rubella virus

RV can establish infection in a variety of human derived cell lines, indicating that the cognate receptors of RV are ubiquitous or exists in various forms [7]. Evidence suggests that the attachment of RV virion to the host cell is mediated through the E1 protein [10]. Besides, the E1 protein plays a crucial role in membrane fusion in the endosomal compartment. This fusogenic activity has been shown to be mediated by the 28 residue internal hydrophobic E1 domain [11]. Once inside the endosome vacuole, the low pH of 6.0 or even lower induces a conformational change in the E1 and E2 glycoproteins, leading to the fusion of the viral envelope with the endosomal membrane [10].

A requirement of host cell components, such as membrane phospholipids and glycolopids, has been shown for the entry of RV into host cells. Further, *N*-acetylglucosamine, glucose and galactose may also participate in the process [12, 13]. Cong et al. [14] showed that one of the host cell receptors identified to bind the E1 protein, is myelin oligodendrocyte glycoprotein (MOG), a member of the immunoglobulin superfamily. MOG is mainly expressed in the central nervous system and its expression has also been detected in other tissues such as spleen, liver and thymus of mice [14]. Due to the restricted expression of MOG in cells of these tissues, the role of other receptors and co-receptors in RV attachment cannot be excluded [14]. The mechanism of the RV entry is thought to be through the endocytic pathway.

## Replication of rubella virus

The replication of the RV involves four distinct viral RNA species (1) single stranded 40S RV genomic RNA (3.8 × 10^3^ kDa), (2) 24S subgenomic RNA (1.2 × 10^3^ kDa) that amounts to the one-third of the genomic RNA in infected cells, (3) viral replicate intermediate of 21S, representing partial double stranded RNA (dsRNA), and (4) viral replicative form of 19 to 20S representing full dsRNA [7]. The single stranded 40S RV genomic RNA serves as a template for the synthesis of 40S negative polarity RNA strand. In addition, it also serves as a messenger of non structural proteins. The 40S negative polarity strand in turn, acts as template for the transcription of both the 40SRNA and the 24S RNA [6]. Nascent 40S RNA is then packaged with RV capsid protein to form the nucleocapsid.

## Translation, processing and assembly

The 24S subgenomic mRNA is translated as polyprotein precursor, which is then translocated into the endoplasmic reticulum (ER) by a specific signal located at the amino terminal of the E1 and E2 protein [7, 15–17]. Within the ER, sequential cleavage of C from E2, and then E1 from E2 occurs. The budding and assembly of rubella viral structures occurs at the Golgi membranes in host cells. The newly budded virions (immature virions) appear as uniformly dense particles within the Golgi complex. These immature virions then undergo structural reorganization during the transit in the Golgi complex, to form mature virions which are secreted to the extracellular environment [18].

## Cytoskeleton changes during rubella infection

The survival of a virus during the course of pathogenesis depends on its ability to manipulate the biological pathways of the host. This is also true for RV, as the genome of the virus codes for only five proteins. Interaction of the RV proteins with the host cell machinery is therefore needed for the survival of the virus and its replication. Several strategies are used by the virus to manipulate the cellular processes of the host in order to multiply efficiently. RV infected cells have been shown to grow and divide slowly in comparison to uninfected cells [19]. Several cell lines infected with RV, cease to grow within a few passages. This phenomenon has been attributed to events such as chromosomal breakage [20] and disruption of actin filaments [21]. Actin forms a part of the cellular cytoskeleton and is known to play a crucial role in cell mitosis. Immunofluorescence studies using antibodies to actin have shown significant alteration in the arrangement of actin fibers during virus infection. Rather than the filamentous actin observed in the uninfected cells, amorphous clumps of fluorescent foci representing depolymerised actin filaments were detected [21]. Thus, disruption of actin assembly during RV infection may result in corresponding inhibition of cell mitosis. Reduced mitotic activity has been shown in congenital infected embryonic primary cell cultures and slowing down of cell division has been reported in RV infected human fetal cells [22, 23].

## Mitochondrial changes during rubella infection

Mitochondria play a crucial role in ATP production and cellular metabolism. In addition to supply ATP for the process of virus replication and assembly, mitochondria serves as a platform for viral replicase complexes in some viruses. For instance, flock house viral RNA polymerase is targeted to mitochondria by an N-terminal sequence motif and is known to replicate in mitochondria [24, 25]. The association of mitochondria and RV infection is further supported by the fact that, the phospholipid cardiolipin that is specific to the inner mitochondrial membrane, was reported to be present in the RV virions [26]. As mentioned above, mitochondria serves as a platform for viral replicase complex, with the clumping of mitochondria around the RV replication complexes [27]. It has been therefore suggested that RV replication is an energy intensive process and therefore, the mitochondria migrates to the vicinity of RV replication complexes to support this [27].

Involvement of mitochondria in RV infection is also suggested by reports of increase in respiration, glycolysis and alanine synthesis during the period of viral adsorption and penetration [28–30]. RV infection results in significant increase in respiratory chain (RC) complex II activity and moderate increase in complex III activity along with decrease in complex IV activity [31, 32]. Also, following RV infection there is an increase in ΔΨ_m_ and high levels of intracellular ATP are observed [31]. It is worth noting that this increase in respiration is achieved without the induction of oxidative stress [32]. Analysis of mRNA expression of the various subunits of the RC complexes shows that, complexes I, III, and IV are slightly expressed while subunits A and B of complex II (succinate dehydrogenase) show the highest rate of induction [32]. Mitochondrial RC is maintained by the coordinated action of the PGC-1 family of co activator, which further controls transcription factors such as NRF1 and NRF2 [33]. While NRF1 regulates the expression of protein required for mitochondrial respiration, NRF2 is needed for the expression of antioxidant enzymes [34]. Both NRF1 and NRF2 are highly expressed in RV infected cells and this possibly explains the increased activity of RC and low level of oxidative stress induction during RV infection [32].

The capsid protein of RV is known to associate with mitochondria. Expression of the capsid protein in the absence of other RV proteins, results in clustering of mitochondria and plaque formation [35]. The RV capsid also binds to p32, a protein known to play a crucial role in many apoptotic pathways and this interaction is required for RV replication [35–37]. The p32 protein is known to interact with several cellular proteins having diverse functions. Although it is predominantly a mitochondrial protein, it has been shown to shuttle between nucleus and mitochondria [38]. During the replication of RV, the p32 protein has been shown to mediate the microtubule mediated trafficking of mitochondria to the replication site in order to meet the energy demands during the replication process [31].

The capsid protein of RV is shown to inhibit the import of p32 protein into mitochondria [39]. Studies suggest that the import of pro-apoptotic proteins to mitochondria may be an integral part of some apoptotic mechanism [40]. As the role of p32 in programmed cell death through multiple mechanisms is well established, it is reasonable to speculate, that RV capsid protein can delay the onset of apoptotic mechanisms, by preventing the translocation of p32 to mitochondria [41–43]. The replication cycle of RV is longer when compared to that of related virus, is represented by long eclipse period with slow replication kinetics and peak virus production does not occur for 48 h [44]. In this context it would be highly desirable if apoptosis were inhibited to facilitate the establishment and maintenance of persistent infections.

## Rubella infection and apoptosis

Apoptosis, the process of programmed cell death, plays a key role in the pathogenesis of many viruses including RV [45, 46]. It has been proposed that RV induced apoptosis may be associated with development of CRS [47]. Although apoptosis of host cells during a virus infection may facilitate the spread of infection to the nearby cells, the apoptosis of host cells during the early phase of virus infection might restrict virus replication efficiency [48]. Induction of apoptosis by RV varies considerably among cell types [47–51]. RV can cause cytopathic effects such as, rounding of cells, and detachment of cells from the monolayer in cell lines such as Vero and BHK 21 [7, 48]. It was shown that RV induces apoptosis in non proliferative primary cultures of cytotrophoblasts and explants of chorionic villi derived from human placenta [52]. However, RV infection failed to induce apoptosis in proliferative human fibroblasts derived from whole embryos of 10 week gestation and fetal lung fibroblasts [52]. Although RV infection is known to induce apoptosis in many cell types, in most cases synthesis of viral proteins and release of virions occurs well in advance of extensive apoptosis [53]. For example, the analysis of the time course of RV infection in Vero cells shows that robust expression of structural proteins is first detected at 16 h post-infection and secretion of infectious virions peaks 32 h later [54]. However, the expression of pro-apoptotic proteins p53 and p21 and late apoptotic events such as DNA fragmentation, are not seen until 5–7 days post-infection, suggesting that apoptosis occurs long after the peak of virus production [49].

It has been suggested that, active replication and the formation of replication complex is required for RV induced apoptosis during acute infection, pointing to the fact that structures found within this complex, such as dsRNA [55, 56] and non structural proteins [57], might play a role in apoptosis. It has been reported that the capsid protein associates with the RV replication complex and this is thought to modulate the activity of the replication complex [58, 59]. However, later transfection studies, involving the expression of RV structural proteins (E1, E2 and capsid) in RK13 cells have shown that the capsid proteins by themselves, can induce apoptosis in the absence of replication [60]. The absence of substantial cell death, in cell lines that only support a low level of RV infection, indicates that accumulation of viral proteins is needed to induce apoptosis [6]. Studies by Ilkow et al. [53] showed that the RV infected cells, are resistant to apoptosis 48 h post infection, when challenged with various apoptotic stimuli. However, the disagreement between the two studies could be due to the following reason. Firstly, in the aforementioned study, the capsid protein was reported to be pro-apoptotic in Rabbit kidney cells (RK-13) but not other cell lines. In the study by Ilkow et al. it was shown that capsid protein blocks apoptosis in multiple cell lines. This result is consistent with the fact, that stable cell lines expressing high levels of RV structural proteins including capsid, is readily established in several cell types [48, 61, 62]. The inhibitory action of capsid protein is mediated by binding to proapoptotic protein Bax before or after it is translocated to mitochondria. Interaction of capsid with Bax induces a conformational change in the Bax protein, leading to formation of hetero-oligomers that are incompetent for pore formation, by which subsequent efflux of cytochrome c from mitochondria is blocked [53]. Besides Bax, a key role is played by the Bcl-2 and Bcl-X_L_ family of proteins in the regulation of apoptosis [50, 63]. In order to determine the role played by these regulatory proteins in RV induced apoptosis, these proteins were transfected in RK13 and BHK21 cells infected with RV. No protection was seen against the apoptosis induced by RV in RK13 cell line. However, increased expression of Bcl-X_L,_ protects cells from apoptosis and it was therefore proposed that the differential response to RV induced apoptosis is a unique property of cells and possibly indicative of how selective organ damage occurs in CRS fetus [50].

RV capsid protein also has been shown to bind to the cellular prostate apoptosis response-4 (par-4) protein [36]. Several studies have highlighted the role of par-4, in sensitizing cells to multiple cytotoxic agents. Its expression in cells also has several effects including depolarization of mitochondria, repression of Bcl2, down regulation of the transcription factor NF-κB and caspase activation of caspase enzyme [64–66]. Further, the proapoptotic role of par-4 is regulated by an extensive network of protein–protein interaction and its localization to the nucleus, appears to be important for apoptosis [64, 67, 68]. Based on these findings it has been speculated that capsid protein can inhibit apoptosis by binding to par-4 in cytosolic compartment and inhibiting its translocation to nucleus [39].

## Gene expression changes during rubella infection

**Fig. 2:**
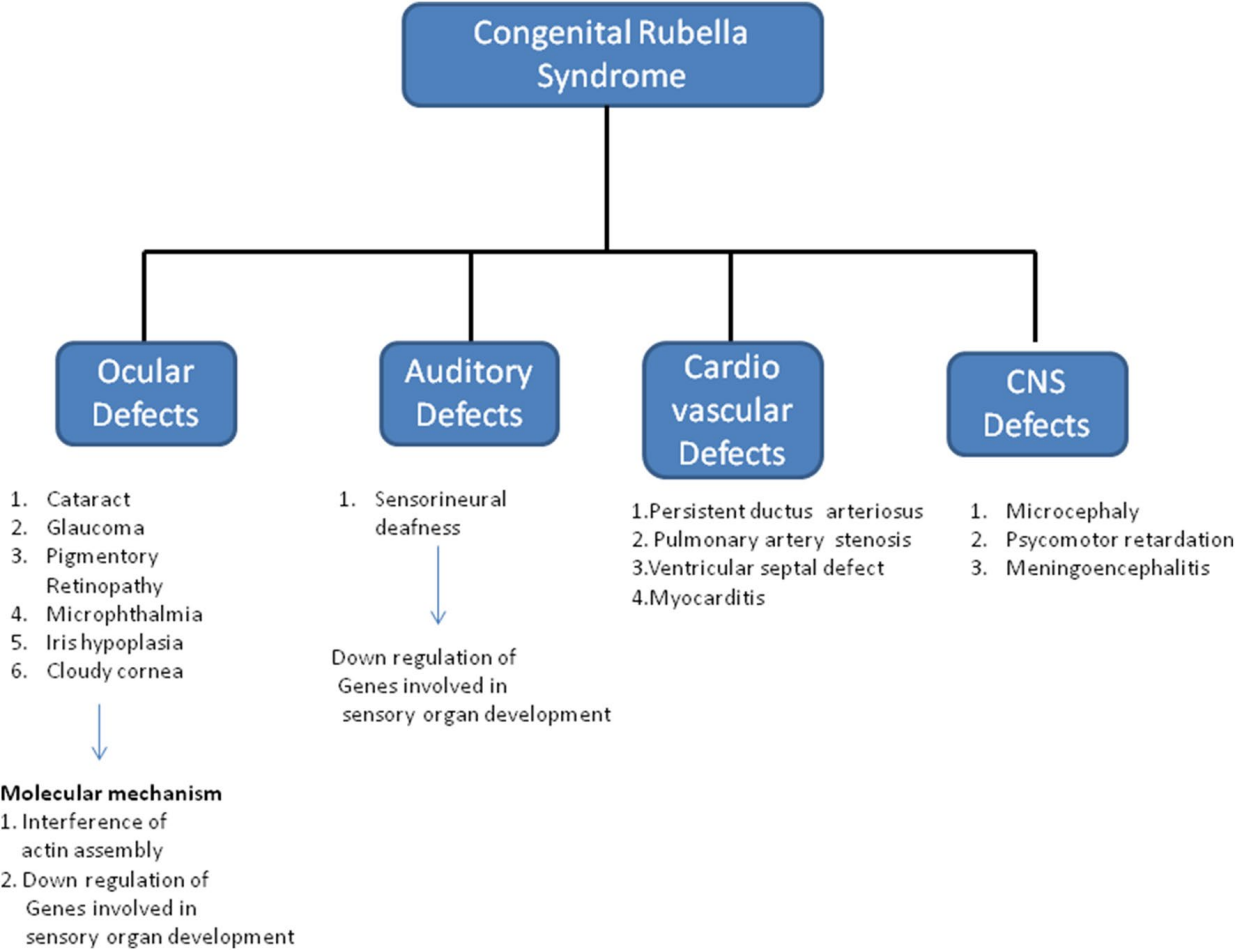
Clinical features of congenital rubella syndrome and molecular mechanism. The molecular mechanisms for ocular and auditory defects have been included, however for cardio vascular and central nervous system defects due to lack of information about the molecular mechanism no details have been provided in the scheme

RV infection during critical stages of organ development in fetus can result in CRS (Fig. 2). Additionally, due to the immature immune system, CRS infants are persistently infected at birth and this is believed to account for the clinical manifestations of CRS. Most of the studies related to CRS are derived from studies on immortal adult cell lines, as discussed above in this review. As these cells are distinct from fetal cells, such studies give limited information regarding the effects of RV infection during embryonic development. However, comparison of the gene expression following RV infection in fetal and adult cells can give novel insights into the molecular mechanism of teratogenicity of RV. Comparison of up and down regulated genes, in primary fetal endothelial cells derived from human umbilical vein (HUVEC) and adult endothelial cells derived from human saphenous vein (HSaVEC), show that the majority of genes expressed in both fetal and adult origin cells, during RV infection are common, however a few unique genes show differential expression [69]. The most remarkable among them are the genes involved in sensory organ development, such as Ceroid-lipofuscinosis neuronal 8 (CLN8), Fibroblast growth factor receptor 2 (FGFR2), Frizzled family receptor 3 (FZD3), Jagged 2 (JAG2), Myosin 7A (MYO7A), Nance-Horan syndrome (NHS), Noggin (NOG), and Solute carrier family 25, member 27 (SLC25A27), which are down regulated in primary cells of fetal origin. It is therefore proposed that virus replication in the fetal endothelium can result in down regulation of genes required for eye and ear development [69].

Studies have shown that primary fetal fibroblast cells do not undergo apoptosis when infected with RV [49]. A similar observation that, RV does not induce apoptosis in primary embryo fibroblast cultures, leads to the suggestion that the absence of apoptosis could promote fetal virus persistence in congenital infection [52]. This observation was further extended to gene chip analysis studies on infected and uninfected primary human fetal fibroblasts (10 weeks gestation) and adult human lung fibroblast [61]. Of the 33,000 human genes inspected, 632 and 512 genes were up regulated or down regulated in RV infected fetal human embryonic fibroblast (HEF) and adult Hs888Lu (a diploid line of human adult lung fibroblasts) cells, in comparison to uninfected control cells. The common set of genes up regulated by infection with RV in fetal and adult cells, was interferon stimulated genes (ISGs) [70]. This result is consistent with a previous report of induction of genes relevant to interferon-regulated pathways in ECV304 cells, a cell line exhibiting both endothelial and epithelial characteristics [71]. Of the upregulated ISGs in RV infected adult Hs888Lu, many of them were known to be associated with induction of apoptosis such as MX [Myxovirus (influenza virus) resistance] genes and OAS (2′,5′-oligoadenylate synthetase) genes (for a complete list of regulated genes refer [61]). Other pro apoptotic upregulated genes have been associated with interferon response (PML, XIAP-associated factor-1, FOXO3A and IL24) [70, 72, 73]. Similar to the adult Hs888Lu cells, upregulation of some pro apoptotic genes such as MX, OAS and IL24 is seen in fetal cells. However, overall fewer pro-apoptotic genes were upregulated in comparison and more antiapoptotic genes were upregulated and fewer downregulated in fetal HEF. Thus the expression and regulation of pro and anti apoptotic genes following infection favoured apoptosis in adult cells whereas in fetal cells apoptosis was not supported [70].

Studies reveal that different organs of foetuses with CRS have reduced cell size and number when compared to the controls, suggestive of mitotic inhibition [6, 7]. Retinoblastoma tumor suppressor protein (Rb), regulates multiple pathways that influence cell proliferation and differentiation, by interacting with several transcription factors and suppress cellular transcription of several essential genes, until the cell is ready to enter the cell cycle [74]. Sequence analysis of the RV non-structural proteins, revealed the occurrence of a functional retinoma tumor suppressor protein binding motif in the RV p90 protein. This protein binds to Rb through the LXCXE motif, both in vitro and in vivo [75]. However, a later study shows, that in spite of elimination of LXCXE-binding site in the Rb, it still retains the ability to actively arrest cells in G1 [74].

Citron-K (CK) kinase is a downstream target of the Rho family small GTPases and is required for the formation of actin structures during cytokinesis [76]. Studies using CK knockout mice and rats, show severely defective neurogenesis and massive apoptosis in the proliferative zones of the developing cortex, resulting in small cerebral cortices [77]. Further, the cortex of CK knockout animals, shows a significant fraction of multinucleate neurons that arise from defective cytokinesis in cortical progenitors [78, 79]. There seems to be some similarity between CK-deficiency associated phenotype and the manifestation of CRS [76]. RV p90 has been shown to interact with CK and co localize to the cytoplasm [76]. Cellular expression of p90 alone disrupts cytokinesis and can arrest a subpopulation of cells in the cell cycle following S phase. A discrete subpopulation of cells containing the tetraploid nuclei were also identified, [76]. In view of the above discussion it has been postulated that RVp90 interaction with CK can interfere with normal functioning of CK.

The teratogenic effect of rubella appears to be direct as well as indirect. Restricted damage to certain organs reflects a varied response of the fetal cells to virus infection. For example, rubella induced apoptosis is seen only in non proliferative and differentiated cells, while apoptosis was not induced in proliferative cells, which would promote viral persistence. The indirect effect of rubella infection includes secretion of interferon and other cytokines by the infected cells that in turn can disrupt growth and proliferation pathways in developing and differentiating cells leading to congenital effects. This is supported by the reports of upregulation of interferons and cytokines in rubella infected human embryo fibroblast [70].

## Conclusion

In conclusion, RV has a small genome and codes for only five proteins. Based on reports through studies on molecular and cell biology, it is evident that several proteins of the host interact with many of these viral proteins to bring about the teratogenic effects. It is interesting to note that some of the host proteins that regulate cell division and cell growth have been found to associate with RV p90. Furthermore replication of the virus in the host cell, directly and indirectly affects the expression of genes involved in the development of sensory organs. Also, there is evidence of cytoskeleton and mitochondrial changes during RV infection. Thus, the lifelong effects that RV inflicts on the developing fetus are cumulative with the outcome arising from the host RV protein interaction.

## Data Availability

Not applicable.

## Abbreviations

RV: rubella virus
CRS: congenital rubella syndrome
RC: respiratory chain
HEF: human embryonic fibroblast
ISGs: interferon stimulated genes
Rb: retinoblastoma tumor suppressor protein
CK: Citon-K kinase
HUVEC: primary fetal endothelial cells derived from human umbilical vein
HSaVEC: adult endothelial cells derived from human sphenous vein
